# Comparative analysis of microRNA expression profiles of adult *Schistosoma japonicum* isolated from water buffalo and yellow cattle

**DOI:** 10.1186/s13071-019-3450-7

**Published:** 2019-05-02

**Authors:** Xingang Yu, Qi Zhai, Zhiqiang Fu, Yang Hong, Jinming Liu, Hao Li, Ke Lu, Chuangang Zhu, Jiaojiao Lin, Guoqing Li

**Affiliations:** 1 College of Veterinary Medicine, South China Agricultural University, Guangzhou, 510642 China; 2 National Reference Laboratory of Animal Schistosomiasis, Key Laboratory of Animal Parasitology of Ministry of Agriculture, Shanghai Veterinary Research Institute, Chinese Academy of Agricultural Sciences, Shanghai, 200241 China

**Keywords:** MicroRNA, *Schistosoma japonicum*, Deep sequencing, Water buffalo, Yellow cattle

## Abstract

**Background:**

Yellow cattle and water buffalo are important natural reservoir hosts and the main transmission sources of *Schistosoma japonicum* in endemic areas of China. The worms from the two hosts have marked differences in general worm morphology and ultrastructure, gene transcription and protein expression profiles.

**Results:**

To investigate microRNAs (miRNAs) involved in the regulation of schistosome development and survival, we compared miRNA expression profiles of adult schistosomes derived from yellow cattle and water buffalo by using high-throughput sequencing with Illumina Hiseq Xten. *Schistosoma japonicum* from water buffalo and yellow cattle yielded 63.78 million and 63.21 million reads, respectively, of which nearly 50% and 49% could be mapped to selected miRNAs in miRbase. A total of 206 miRNAs were identified, namely 79 previously annotated miRNAs of *S. japonicum* and 127 miRNAs that matched with the *S. japonicum* genome and were highly similar to the annotated miRNAs from other organisms. Among the 79 miRNAs, five (sja-miR-124-3p, sja-miR-219-5p, sja-miR-2e-3p, sja-miR-7-3p and sja-miR-3490) were significantly upregulated in the schistosomes from water buffalo compared with those from yellow cattle. A total of 268 potential target genes were predicted for these five differentially expressed miRNAs. Eleven differentially expressed targets were confirmed by qRT-PCR among 15 tested targets, one of which was further validated through dual-luciferase reporter assay. Among the 127 ‘possible’ *S. japonicum* miRNAs, ten were significantly differentially expressed in the schistosomes from these two hosts.

**Conclusions:**

These results highlight the important roles of miRNAs in regulating the development and survival of schistosomes in water buffalo and yellow cattle and facilitate understanding of the miRNA regulatory mechanisms in schistosomes derived from different susceptible hosts.

**Electronic supplementary material:**

The online version of this article (10.1186/s13071-019-3450-7) contains supplementary material, which is available to authorized users.

## Background

Schistosomiasis, one of the most severe zoonotic diseases caused by several species of blood flukes of the genus *Schistosoma*, affects approximately 260 million people worldwide [1]. Schistosome infections in individuals can lead to severe ascites, hepatosplenomegaly, hepatic fibrosis and even death [2]. The three main species causing human disease are *Schistosoma japonicum*, *Schistosoma mansoni* and *Schistosoma haematobium* [3]. Among them, *S. japonicum* is prevalent mainly in China, the Philippines and some areas of Indonesia [4]. Although schistosomiasis control in China has been remarkably successful through the promotion of synchronous control strategies between humans and domestic animals over the past six decades [5], there were still 54,454 human infections of schistosomiasis by the end of 2016 [6]. The control of schistosomiasis relies heavily on chemotherapy with Praziquantel, the only widely applied drug [7]. However, the extensive use of this drug has led to major concerns regarding the development of drug resistance, which would severely compromise current treatment and control efforts.

*Schistosoma japonicum* has a complex life-cycle consisting of several different stages of development (the egg, miracidium, sporocyst, cercaria, schistosomulum and adult stages) and it requires a transformation in mammals (definitive hosts) and *Oncomelania* snails (intermediate hosts) [8]. Forty-six species of mammals have been reported to carry natural infection with *S. japonicum*, such as pigs, rabbits, rats, dogs, cats, horses, cattle, goats and monkeys [9]. The susceptibility of different hosts to *S. japonicum* varies. Previous studies have revealed that mice, goats and yellow cattle are more susceptible than rats and water buffalo to *S. japonicum* infections [10]. Among mammalian hosts, domestic animals, particularly yellow cattle and water buffalo (*Bos taurus* and *Bubalus bubalis*), are the most important natural reservoir hosts and the main transmission sources of *S. japonicum* in endemic areas of China [11, 12]. Because they graze freely in areas near water (streams, marshes and lake regions), these hosts, compared with humans and other animal hosts, are able to excrete at least 100 times more daily fecal output into surrounding areas [12, 13].

Yellow cattle are better suited than water buffalo for the development of *S. japonicum* [10]. The worm length, worm recovery rate, female worm egg production and percentage of paired worms derived from yellow cattle are significantly greater than those in water buffalo [14]. In addition, the surface topography and internal structures at the ultrastructural level have also been found to differ between adult worms isolated from water buffalo and from yellow cattle [15]. Recent studies in our laboratory have revealed that adult *S. japonicum* from yellow cattle and water buffalo displays different expression profiles at the transcript and protein levels [15, 16]. The expression of some functional proteins is closely correlated with the development of schistosomes, and is required for growth, reproduction, energy metabolism and evasion of the host immune response. In general, schistosomes from yellow cattle and water buffalo exhibit marked differences in developmental rates and substantial morphological changes during infection, and subtle gene regulation may be required to carry out the related complex biological processes [17].

miRNAs are small noncoding RNAs that play important roles in post-transcriptional gene regulation through translational inhibition and destabilization of their mRNA targets [18]. They exert important roles in various biological processes, such as development [19], cell proliferation and differentiation [20], cell apoptosis [21], metabolism [22] and signal transduction [23] in many organisms. In recent years, large numbers of miRNAs have been identified from schistosomes, and accumulating evidence suggests that schistosome miRNAs play important roles in regulating worm growth, sexual maturation (sja-miR-1 and sja-miR-7-5p) and development (sja-miR-36-3p) [19, 24, 25], as well as in host-parasite interactions (bantam and sja-miR-10) [26]. In addition, several lines of evidence also suggest that miRNAs may regulate the pathogenesis of schistosomiasis (sja-bantam) [27]. Previous research from our group has shown that some schistosome miRNAs are differentially expressed among different susceptible rodent hosts [28, 29]. However, the profiles of miRNA expression in *S. japonicum* from natural host water buffalo and yellow cattle are unknown.

Here, we performed the first deep sequencing study to obtain miRNAs profiles of adult *S. japonicum* from water buffalo and yellow cattle. Our results provide valuable information for understanding the regulatory roles of miRNAs involved in the development and survival of *S. japonicum* derived from water buffalo and yellow cattle, and lay a solid foundation for further functional studies of these differentially expressed miRNAs.

## Methods

### Animal challenge and worm collection

Four male water buffalo and four male yellow cattle (15–19 months-old) were purchased from schistosome non-endemic areas and were treated with albendazole and ivermectin to eliminate parasitic helminths 30 days before the study. All animals had similar body weights and were raised by trained animal keepers. *Schistosoma japonicum* (Chinese strain) cercariae were obtained from the National Reference Laboratory of Animal Schistosomiasis at the Shanghai Veterinary Research Institute. The water buffalo and yellow cattle were each challenged percutaneously with 1000 cercariae of *S. japonicum* administered to the upper back through the cover glass method [15, 30].

All infected animals were sacrificed 8 weeks post-infection, and the adult worms were perfused from the hepatic portal vein. After being washed with phosphate-buffered saline, equal amounts of parasites were divided into several sterile cryopreservation tubes and stored in RNAlater (Ambion, Carlsbad, CA, USA). The worms were then stored at − 80 °C until further use.

### Total RNA isolation, small RNA library construction and sequencing

RNA was extracted from the *S. japonicum* (approximately ten pairs) derived from four water buffalo and four yellow cattle, respectively, with Trizol (Invitrogen, Carlsbad, CA, USA) and further purified with an RNeasy mini kit (Qiagen GmbH, Hilden, Germany) according to the manufacturer’s instructions. The RNA integrity and quality were verified with an Agilent 2200 bioanalyzer (Agilent Technologies, Inc., Santa Clara, CA, USA) with an RIN (RNA integrity number) > 6.0. Small RNAs of 18–35 nt in length were used to construct the sequencing libraries using a NEBNext^®^ Small RNA Library Prep Set for Illumina (New England Biolabs, Beverly, MA, USA). Briefly, these small RNAs were ligated with 5′-RNA and 3′-RNA adapters, and were reverse transcribed into cDNAs. Then an indexed PCR was performed. The PCR products were size selected, purified and sequenced by HiSeq Xten (Illumina, San Diego, CA, USA). These data have been deposited in the Gene Expression Omnibus database (GEO; http://www.ncbi.nlm.nih.gov/projects/geo/), with the GEO Series accession number GSE124351.

### Data processing and bioinformatics analysis

After deep sequencing, the raw sequencing data were evaluated in FAST-QC software (http://www.bioinformatics.babraham.ac.uk/projects/fastqc/), including the quality distribution of nucleotides, position specific sequencing quality, GC content, proportion of PCR duplication and k-mer frequency. Reads that matched to ribosomal RNA (rRNA), transfer RNA (tRNA), small nuclear RNA (snRNA), small nucleolar RNA (snoRNA) and other non-coding RNA (ncRNA) sequences previously deposited in Rfam 8.0 and NCBI GenBank were excluded. Raw data were further processed to remove low quality reads, adaptor sequences and contaminant reads. First, we applied read filtering to the raw reads to achieve clean data according to the following criteria: (i) 30% of bases with a Phred quality score < 20; (ii) read length < 17 bps; and (iii) adaptor sequence. Then, the clean data were mapped to the *S. japonicum* genome [31] (WormBase ParaSite v.10; http://parasite.wormbase.org/index.html) [32, 33] with the Burrows–Wheeler alignment (BWA) tool [34] to remove contaminant reads from the host *Bos taurus* transcriptome. Reads mapping to the genome were for *S. japonicum* miRNA identification with BWA-ALN (bwa aln -n 0.04 -e 3 -l 32 -k 2 -t 8) and mapping to the miRbase v.22.0 database (http://www.mirbase.org/) (miRBase. 22.0; released March 2018) to obtain known miRNAs and their expression levels, and only reads that mapped to mature known miRNA regions were calculated as the known miRNA raw counts.

Furthermore, novel miRNAs were predicted by exploring the Dicer cleavage sites, minimum free energy and secondary structure of the former unmapped reads that were mapped to the *S. japonicum* genome but not to the known miRBase database of *S. japonicum* in the software miRDeep2 [35]. Then, these reads were utilized to map to the predicted miRNA precursor sequences with BWA-ALN. The expression levels of the novel miRNAs were calculated according to the reads mapping to the mature regions of the precursor miRNA sequences. The predicted mature novel miRNAs were blasted [36] against the miRNA sequences of the related species. Secondary structure prediction of individual miRNAs was performed in MFOLD software (v.2.38, http://mfold.rna.albany.edu/?q=mfold/RNA-Folding-Form). Before analysis of differentially expressed miRNAs, weakly expressed miRNAs were filtered according to the following criteria: (i) the counts of the miRNAs were greater than 5 in at least one sample, (ii) the sums of the counts of the miRNAs were greater than 10 in all the samples.

High confidence miRNAs were achieved and the EdgeR algorithm [37] was applied to calculate the differentially expressed miRNAs of known miRNAs and novel miRNAs separately based on the raw counts data achieved above normalized by weighted trimmed mean of M-values (TMM). In order to discover significantly differentially expressed miRNAs, fold change (water buffalo/yellow cattle) analyzed by TMM normalized data and *P*-values calculated by negative binomial model were subjected to the following criteria: (i) log_2_^Fold Change^ > 0.585 or < − 0.585; and (ii) *P*-value < 0.05. The workflow of sequencing data analysis is illustrated in Additional file 1: Figure S1. The output from mirDEEP2 for miRNA prediction is shown in Additional file 2: Table S1.

For the authenticity and reliability consideration of miRNAs, the identified miRNAs were classified into three levels: (i) known *S. japonicum* miRNAs that had been annotated in miRBase; (ii) miRNAs that matched with the *S. japonicum* genome and the annotated miRNAs of *S. mansoni*; (iii) miRNAs that matched with the *S. japonicum* genome and annotated miRNAs of other species such as *Sus scrofa*, *Rattus norvegicus*, *Bos taurus*, *Mus musculus* and *Homo sapiens*. miRNAs expressed in more than three worm samples in both the yellow cattle group and water buffalo group were regarded as novel miRNAs.

### Prediction of target genes and functional annotation of differentially expressed miRNAs

Miranda (http://www.microrna.org/) and RNAhybrid [38] were used for predicting differentially expressed miRNA targets in mRNAs (5′-untranslational regions (UTR), coding sequence (CDS) or 3′-UTR) [17]. Gene sequences were downloaded from the NCBI website and WormBase ParaSite v.10. The predicted target genes were further mapped and filtered to the correlated differentially expressed genes between adult schistosomes from water buffalo and yellow cattle, which were obtained through oligonucleotide microarray analysis in our previous study (GEO accession no. GSE24615) [15].

Gene ontology (GO) analysis was performed to study the biological implications (the cellular components, molecular functions and biological process) of the target genes of the differentially expressed miRNAs [39]. Kyoto Encyclopedia of Genes and Genomes (KEGG) pathway analysis was used to determine the significant pathways of the differentially expressed genes [40]. Fisher’s exact test was applied to identify the significant GO and pathway terms (*P* < 0.05).

### Quantitative reverse transcription PCR (qRT-PCR) based expression analysis of miRNAs and their predicted targets

Five differentially expressed known miRNAs were selected for analysis with qRT-PCR with SYBR green. Total RNA from ten pairs of adult worms was extracted using an miRcute miRNA isolation kit (Tiangen, Beijing, China) according to the manufacturer’s instructions. RNA templates of parasites used for qRT-PCR were obtained from the same worm samples used for sequencing. Total RNA from worms was quantified with a NanoDrop 2000 spectrophotometer (Thermo Fisher Scientific, Waltham, MA, USA) and was reverse transcribed to cDNA with a miRcute Plus miRNA first-strand cDNA synthesis kit (Tiangen). qRT-PCR was performed on an ABI 7500 Real-Time PCR System (Applied Biosystems, Foster City, CA, USA) with an miRcute Plus miRNA qRT-PCR Detection Kit (SYBR Green; Tiangen). Twenty-microliter qRT-PCR reactions contained the following: 10 μl of 2× miRcute Plus miRNA Premix (with SYBR&ROX), 0.8 μl of forward and reverse primer mixtures, 2 μl of ten-fold serially diluted miRNA first-strand cDNA and 7.2 μl of RNase-free water. The procedure for the PCR was: 95 °C for 15 min; 94 °C for 20 s, 64 °C for 30 s, 72 °C for 34 s, five cycles; and 94 °C for 20 s, 60 °C for 30 s, 45 cycles. All assays were performed in triplicate. The 2^−ΔΔCt^ method for relative quantification of gene expression was used to determine the level of miRNA expression, and U6 RNA [25, 41, 42] was used for normalizing the data. The primer sequences are shown in Additional file 3: Table S2.

The potential target genes that might be regulated by these differentially expressed miRNAs were investigated by qRT-PCR. Total RNA was extracted from the same worm samples by using TRIzol^®^ reagent (Invitrogen) according to the manufacturer’s protocol. The quality of total RNA samples was quantified with a NanoDrop 2000 spectrophotometer. Samples were placed in isopropanol and stored at − 80 °C before complementary DNA (cDNA) was synthesized by using a Super Script™ III Reverse Transcriptase kit (Invitrogen). PCR amplification was performed with a SYBR^®^ PremixEx Taq™ kit (TaKaRa, Dalian, China) on an ABI 7500 Real-Time System (Applied Biosystems). The reaction conditions were as follows: 95 °C for 30 s, 40 cycles of 95 °C for 5 s and 60 °C for 34 s. *Sj*NADH was selected as an internal reference [16]. The target gene numbers and primer sequences are shown in Additional file 4: Table S3.

### Dual-luciferase reporter assay

On the basis of the sequences in Wormbase of the 11 potential targets confirmed by qRT-PCR, fragments of one gene (Sjp_0008430, Hsc20) that contained the putative binding site for sja-miR-219-5p were amplified by PCR. Two primers, H20E (5′-GCT AGC CGG TAG TCG TAC ATG CTG GAA C-3′) with *Nhe*I restriction site (underlined) and H20F (5′-CTC GAG GCC TCC AAG CTA ATT CAT GTT CTC-3′) with *Xho*I restriction site (underlined), were designed on the basis of the *S. japonicum* gene (Sjp_0008430) sequence. The PCR products were purified and ligated into vector pMD19-T (TaKaRa) plasmid. The pMD19-T-Sjp_0008430 plasmid was transformed into Trans109 competent cells (TransGen Biotech, Beijing, China). After selection of cells on fresh LB agar plates supplemented with 100 mg/ml of ampicillin, the plasmid was purified with a Plasmid Mini Kit (Tiangen). The purified constructs were further digested with *Nhe* I/*Xho* I (TaKaRa) and then cloned into a pmirGLO luciferase vector (Promega, Madison, WI, USA). After confirmation through *Nhe*I/*Xho*I digestion and sequencing by Sangon Biotech (Shanghai, China), positive clones were named pmirGLO-Sjp_0008430.

sja-miR-219-5p mimics or inhibitors and their corresponding controls were chemically synthesized by GenePharma Inc. (Shanghai, China). Luciferase reporter constructs together with mimics or inhibitors or negative control and inhibitor control (Additional file 5: Table S4) were then transfected into human embryonic kidney 293T (HEK 293T) cells in 12-well plates. The system contained 1.6 μg of luciferase plasmid, 40 pM of mimics or inhibitors and their negative controls. Luciferase activity was measured using the Dual-Luciferase Reporter Assay System (Promega) after transfection for 48 h. Both *Renilla* luciferase activity and firefly luciferase activity were measured, and the relative luciferase activity was normalized to the *Renilla* luciferase activity [43]. Each experiment was repeated at least three times.

### Statistical analysis

Data are expressed as the mean ± standard deviation. Differences between groups were determined by Student’s t-test. A *P*-value < 0.05 was considered to indicate a statistically significant difference.

## Results

### Overview of small RNA library analysis by deep sequencing

**Fig. 1:**
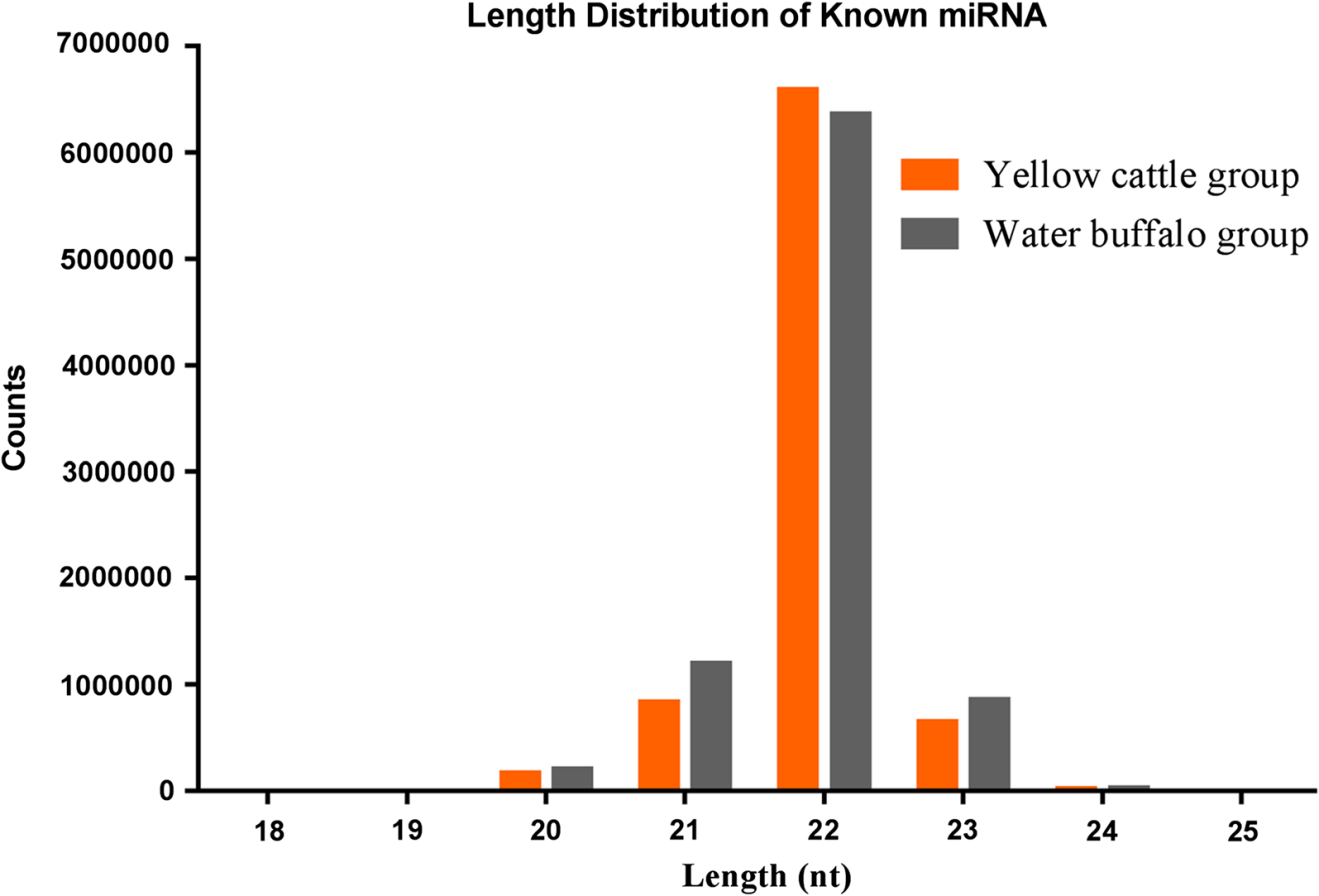
Length distribution of known miRNAs of adult *S. japonicum* from water buffalo and yellow cattle

**Fig. 2:**
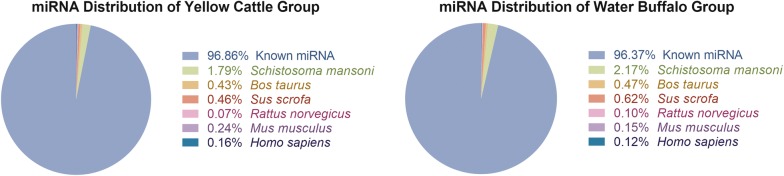
Preliminary analysis of the reads of *Schistosoma* from yellow cattle and water buffalo. 96.86% and 96.37% of worm miRNAs counts derived from yellow cattle and water buffalo, respectively, were identified as *S. japonicum* known mature miRNAs sequence. The distribution features of miRNAs counts from other organisms is also described

A total of 126,989,624 reads were obtained, with an average of 15,873,703 valid reads per sample. The adult *S. japonicum* from water buffalo and yellow cattle yielded 63.78 million and 63.21 million reads, respectively. The obtained clean reads were mapped to the *S. japonicum* genome (WormBase ParaSite v.10.) with an average mapping ratio of 99.62%. Among them, the mapped rate of the worms in water buffalo samples ranged between 99.65–99.75%, whereas that in the yellow cattle ranged between 99.12–99.70%. The mapped reads were then used for miRNA identification and prediction. The length distribution of the known miRNAs from the eight sequencing libraries mainly ranged from 18 to 24 nt (Fig. 1). A total of 96.86% and 96.37% of worm miRNAs derived from yellow cattle and water buffalo, respectively, were identified as *S. japonicum* known mature miRNAs sequence in the miRbase v.22.0 database (Fig. 2).

### Comparison of the expression of annotated *S. japonicum* miRNAs between adult schistosomes from water buffalo and yellow cattle

**Fig. 3:**
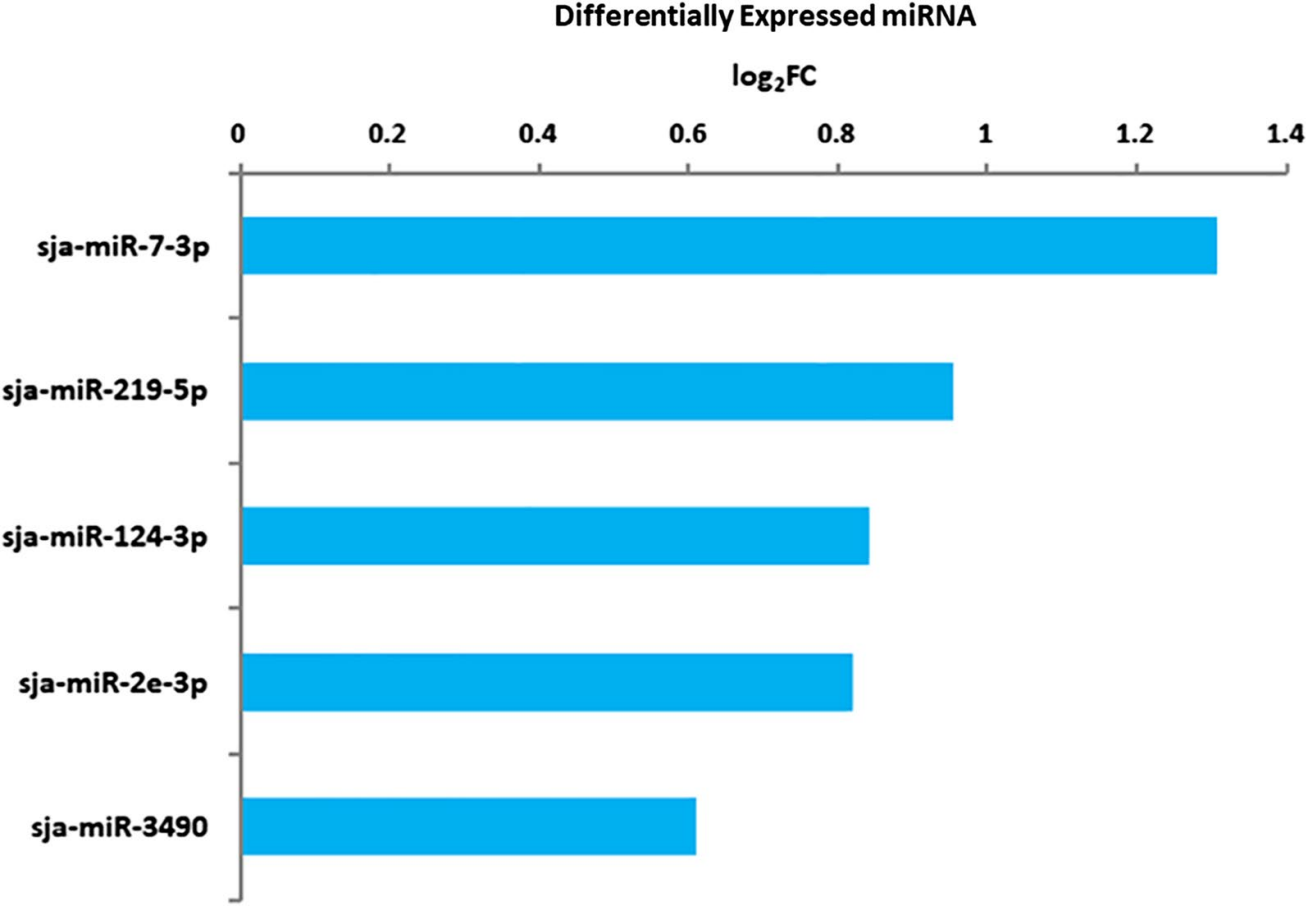
The significantly differentially expressed miRNAs in adult *S. japonicum* from water buffalo normalized to those in *S. japonicum* from yellow cattle

The miRNA profile data of the adult schistosomes from water buffalo were normalized against those from yellow cattle. The reads that aligned to the genome of *S. japonicum* were further blasted against the miRNA precursor sequences in the miRNA database (miRBase v.22.0) of *S. japonicum*. The 79 previously annotated miRNAs of *S. japonicum* in miRBase were all found but showed different degrees of expression in worms from four water buffalo *versus* four yellow cattle (Additional file 6: Table S5). Among them, sja-miR-125b was most abundantly expressed in the worms of both yellow cattle and water buffalo, whereas sja-miR-3484-3p was excluded from further analysis because its expression was very low. Notably, 5 of the remaining 78 miRNAs were significantly upregulated (log_2_^Fold Change^ > 0.585 or < − 0.585; *P* < 0.05) in the schistosomes from water buffalo compared with those from yellow cattle (Fig. 3 and Additional file 7: Table S6).

### Preliminary identification of novel miRNAs

A total of 127 miRNAs were newly discovered through bioinformatic analysis (Additional file 8: Table S7). The reads that were mappable to the *Schistosoma* genome but did not further map to the known miRNAs of *S. japonicum* in miRbase were used for novel miRNAs prediction with the miRDeep algorithm. Through this process, 127 novel miRNAs were preliminarily confirmed including 66 *S. mansoni* annotated miRNAs and 61 annotated miRNAs from other organisms, such as *Mus musculus*, *Bos taurus*, *Sus scrofa* and *Homo sapiens* (Additional file 9: Table S8). The information related to the precursor miRNA of each predicted miRNA is shown in Additional file 2: Table S1. These 127 novel miRNAs all were found in worms derived from both the yellow cattle and water buffalo groups, although with varying expression levels. Among them, ten novel miRNAs were significantly differentially expressed, and six were upregulated in worms from water buffalo compared with worms from yellow cattle (Additional file 10: Table S9). Five novel miRNAs were first characterized from the 127 novel miRNAs in this study; their locations and sequence characteristics are summarized in Additional file 11: Table S10. The secondary structures of the precursors of the five novel miRNAs are shown in Additional file 12: Figure S2.

### Verification of the five differentially expressed known miRNAs by using qRT-PCR

**Fig. 4:**
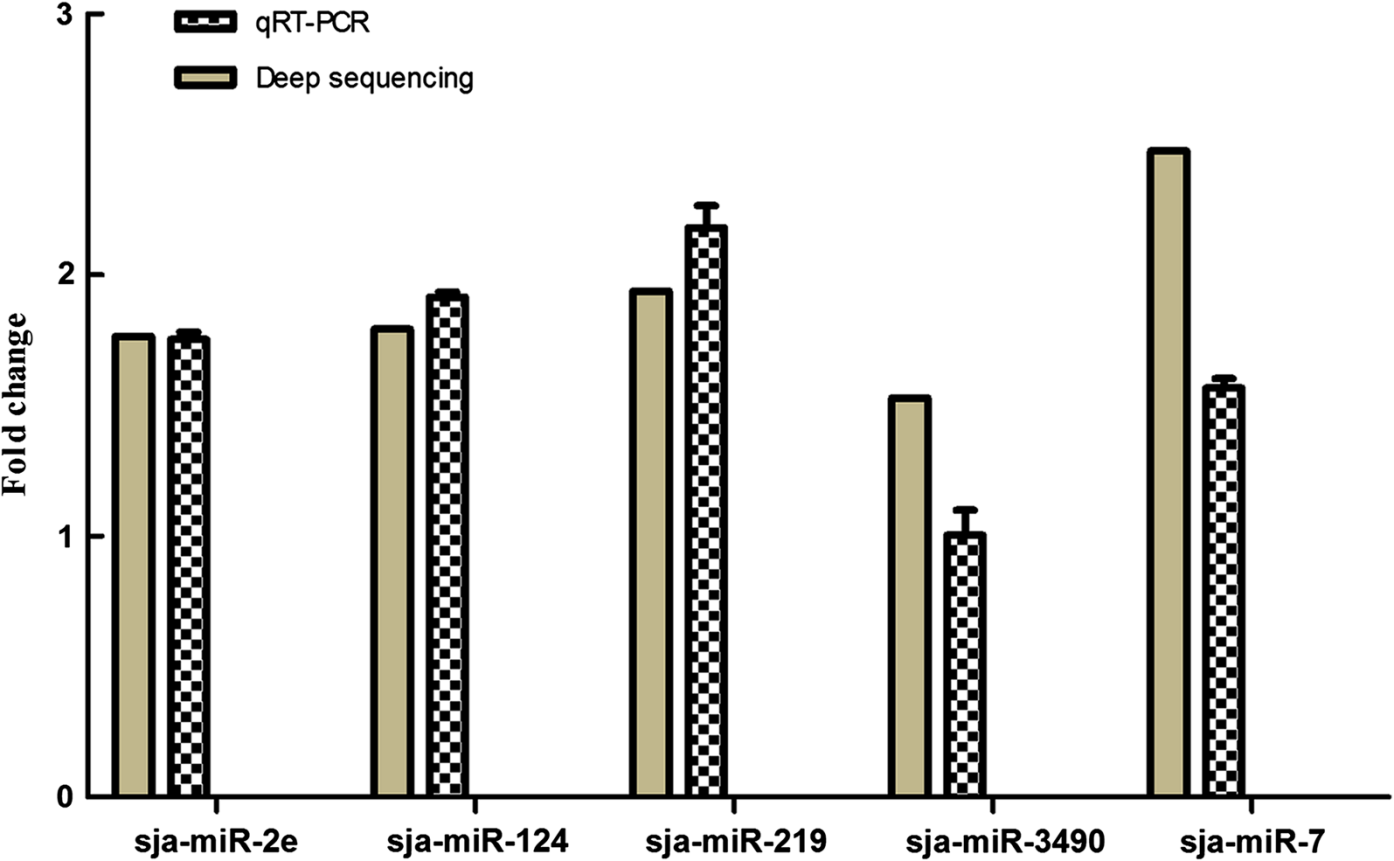
Relative expression of the five miRNAs in *S. japonicum* from water buffalo compared with those in yellow cattle using qRT-PCR analysis. The data are presented as the mean and standard error of the mean, derived from triplicate experiments

Five differentially expressed miRNAs and the endogenous control U6 RNA were assayed by qRT-PCR to verify the deep sequencing profiles. The results showed that four of the miRNAs showed the same expression patterns as those of the deep sequencing results, except that sja-miR-3490 showed a similar expression level, thus suggesting that the high-throughput sequencing results were reliable (Fig. 4).

### Prediction of the target genes for five differentially expressed known miRNAs

A total of 272 potential miRNA-mRNA target relationships of the five differentially expressed miRNAs were found by using miRanda, including 14 for sja-miR-7-3p, 117 for sja-miR-3490, 70 for sja-miR-124-3p, 28 for sja-miR-219-5p and 43 for sja-miR-2e-3p (Additional file 13: Table S11). Among them, sja-miR-2e-3p and sja-miR-7-3p jointly regulate Sjp_0037360, and sja-miR-124-3p and sja-miR-3490 co-regulate Sjp_0037360, Sjp_0022760 and Sjp_0017090; thus, 268 targets were finally predicted.

### Functional enrichment analysis of the predicted targeted genes

**Fig. 5:**
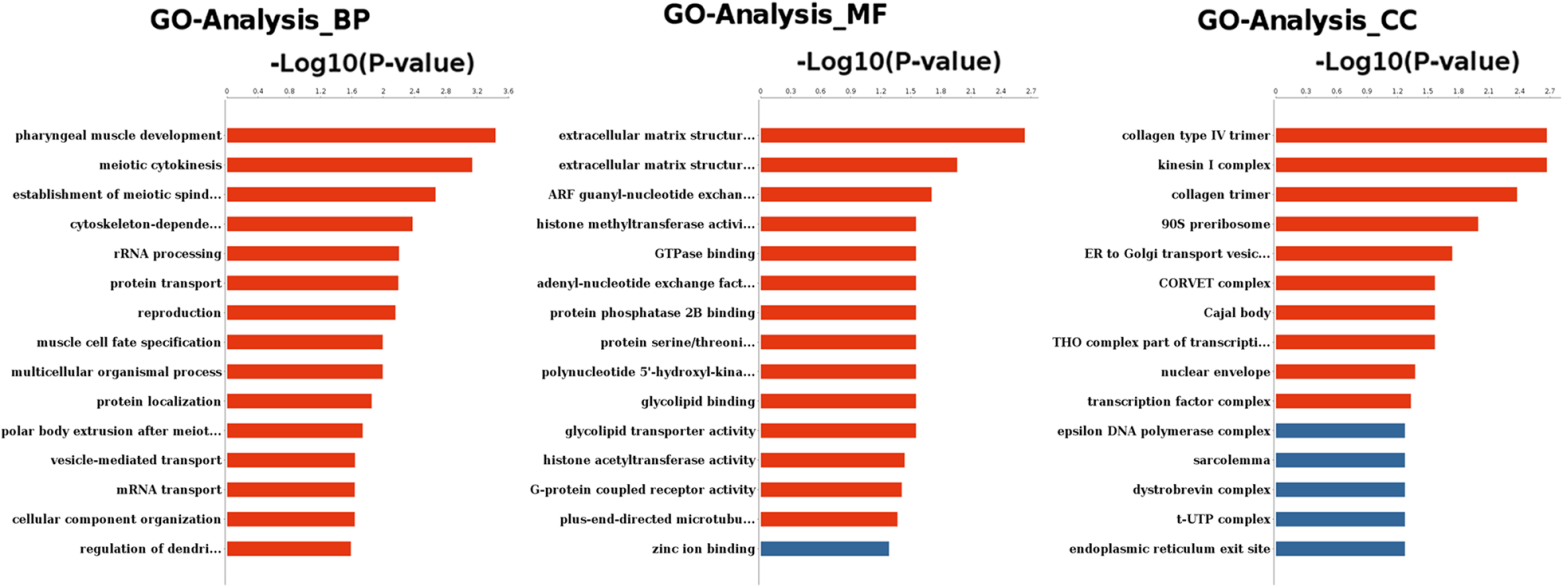
GO analysis of target genes of differentially expressed miRNAs in adult *S. japonicum* from water buffalo and yellow cattle. Targets were classified into three main categories: biological process, molecular function, and cellular component

After target prediction, transcripts were subjected to BLASTX analysis, and the targets included the annotations of septin-7.1, eukaryotic initiation factor 4A, Ras GTPase, putative tropinone reductase, cytohesin-2 and NEM-sensitive fusion protein 2. GO analysis was applied to analyze the main functions of the differentially expressed genes. The results showed that the targets of the differentially expressed miRNAs were primarily involved in pharyngeal muscle development, meiotic cytokinesis, establishment of meiotic spindle localization, cytoskeleton-dependent intracellular transport, rRNA processing, protein transport, reproduction and muscle cell fate specification (Fig. 5 and Additional file 14: Table S12). In the case of cellular components, these genes were classified into 111 categories, of which the top enrichments were related to collagen type IV trimer, kinesin I complex, collagen trimer, 90S pre-ribosome, endoplasmic reticulum to Golgi transport vesicle membrane, nuclear envelope and transcription factor complex (Fig. 5 and Additional file 15: Table S13). On the basis of molecular function, most targets were correlated with extracellular matrix structural constituent conferring tensile strength, extracellular matrix structural constituent, ADP-ribosylation factor (ARF) guanyl-nucleotide exchange factor activity, histone methyltransferase activity (H3-K9 specific) and GTPase binding (Fig. 5 and Additional file 16: Table S14).

**Fig. 6:**
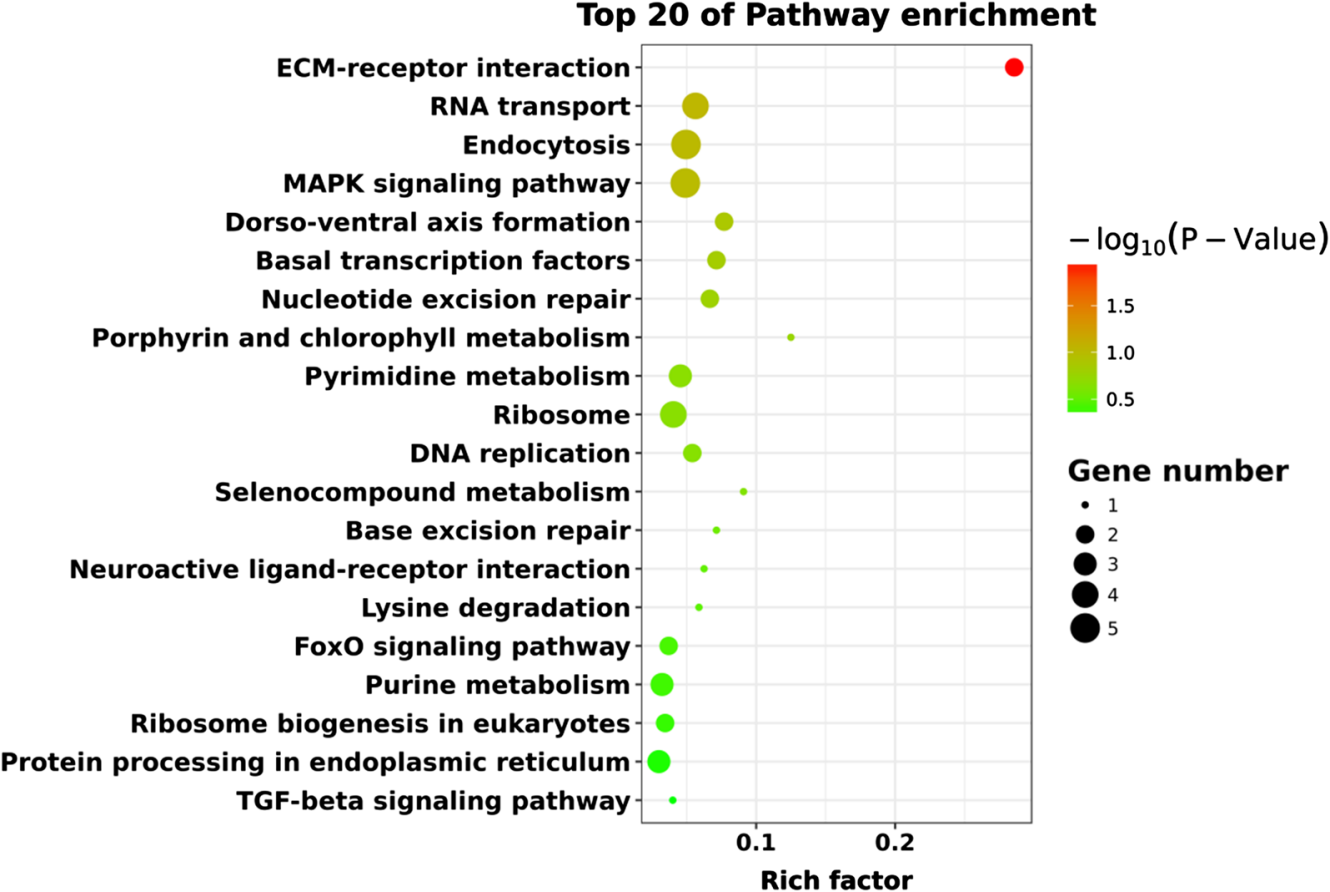
KEGG analysis with the 20 most enriched pathways. The coloring of the − log_10_^( *P*-value)^ indicates the significance of the enriched factor; the circle indicates the target genes that are involved, and the size is proportional to the gene number

KEGG pathway and enrichment analysis indicated that these differentially genes were highly enriched in extracellular matrix (ECM)–receptor interaction, RNA transport, endocytosis, MAPK signaling pathway, dorso-ventral axis formation, basal transcription factors, nucleotide excision repair, porphyrin and chlorophyll metabolism, pyrimidine metabolism, ribosome and DNA replication (Fig. 6 and Additional file 17: Table S15). ECM-receptor interaction (two genes) was the most significantly enriched category with respect to the rich factor and was followed by RNA transport (four genes), endocytosis (five genes) and mitogen-activated protein kinase (MAPK) signaling pathway (five genes). The predicted KEGG pathways provide information about the functions of the miRNAs targets.

### Verification of the target genes by qRT-PCR

**Fig. 7:**
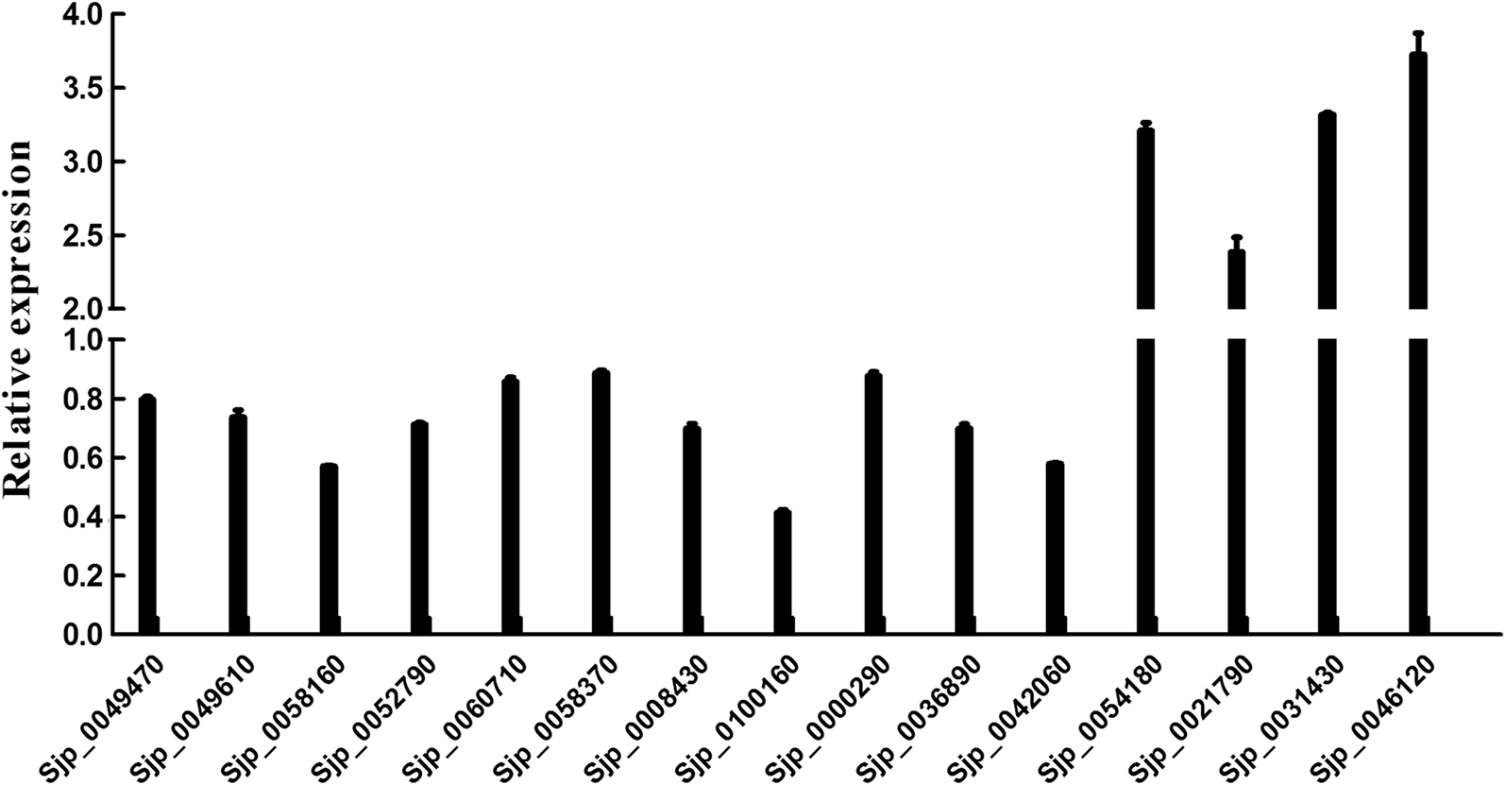
Expression levels of the target genes in *S. japonicum* from water buffalo compared with those from yellow cattle, as revealed by qRT-PCR

Among the 268 predicted targets for the five differential expressed miRNAs, 30 genes mapped to the differentially expressed genes identified in our previous study through a *S. japonicum*-specific microarray analysis. Subsequently, 15 of the 30 differentially expressed genes were randomly selected and further subjected to qRT-PCR verification. The results showed that 11 target genes were downregulated in water buffalo-derived worms compared with those from yellow cattle, whereas the other four genes were upregulated in worms from water buffalo (Fig. 7).

### Validation of miRNA targets by luciferase reporter assays

**Fig. 8:**
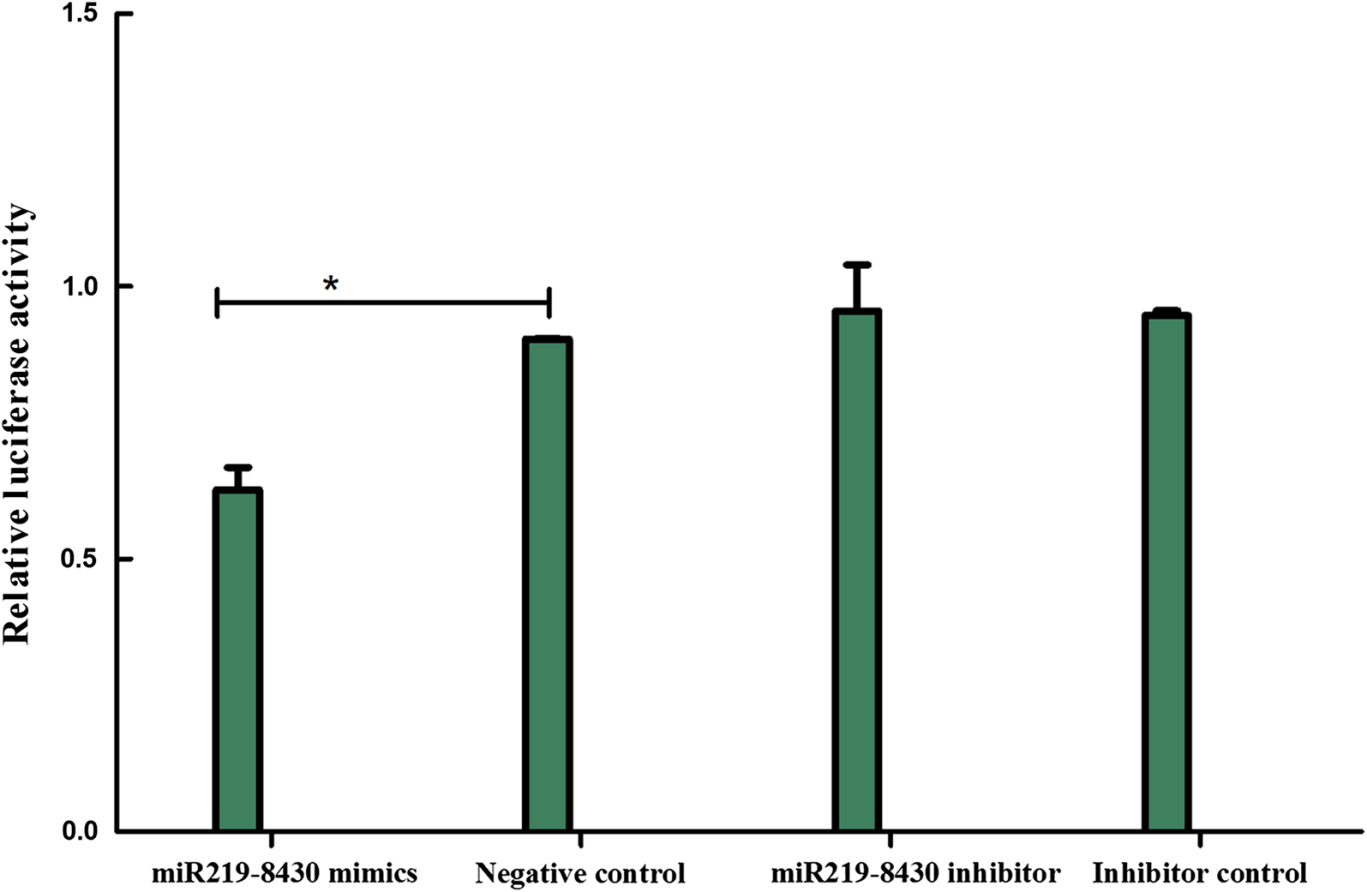
Verification of predicted schistosome miRNA targets (Hsc20) with sja-miR-219-5p in HEK 293T cells

The predicted target gene Sjp_0008430 (Hsc20) was selected for further verification of regulation by sja-miR-219-5p through binding to the complementary region in luciferase reporter assays. The results showed significant changes in luciferase activity in the pmirGLO-Sjp_0008430 plasmid plus sja-miR-219-5p mimics group. As shown in Fig. 8, transfection of sja-miR-219-5p mimics resulted in lower luciferase activity than did transfection with the negative control, suggesting that sja-miR-219-5p downregulates the expression of Sjp_0008430 in a heterologous system.

## Discussion

miRNAs are a class of small regulatory RNAs involved in the regulation of many biological processes through sequence-specific inhibition of target gene expression [44]. *Schistosoma japonicum* has a complex life-cycle consisting of seven developmental stages, with a unique repertoire of gene expression at different stages [8, 45], thus suggesting that gene expression of this parasite is regulated accurately and precisely. Previous studies have revealed that some miRNAs are differentially expressed among the developmental stages and genders of schistosomes [8, 19, 24, 25]. For example, sja-miR-71b-5p, sja-miR-1, sja-miR-36-3p and miR-124-3p are most abundant in the egg, which is the critical mediator of severe pathologic damage in schistosomiasis [46]. sja-miR-71 and sja-bantam expression peak in the cercaria stage, then decline quickly and reach a nadir in the schistosomulum stage [8]. In lung-stage schistosomula, compared with cercariae, sja-bantam, sja-miR-1, sja-miR-124-3p, sja-miR-2a-3p, sja-miR-3492 and sja-miR-36-3p are downregulated [19]. In adult schistosomes, sja-miR-7-5p, sja-miR-61, sja-miR-219-5p and sja-miR-124-3p are dominant in male worms, whereas sja-bantam, sja-miR-71b-5p and sja-miR-3479-5p are predominantly found in female worms [19]. Furthermore, suppression of bantam and miR-31, which are highly expressed in female adult worms, leads to morphological alterations in the ovaries [24]. These studies have broadened the understanding of miRNAs as important regulators in schistosome development, sexual differentiation, maintenance and pathological damage. Further studies on miRNAs may improve understanding of the developmental mechanism of this parasite.

Some miRNAs of *S. japonicum* obtained from different susceptible hosts have different expression profiles and expression levels. Forty-one miRNAs have been found to be differentially expressed in ten-day-old schistosomula isolated from *S. japonicum* non-susceptible host Wistar rats and susceptible host BALB/c mice, including four miRNAs with specific functions in either schistosome development (sja-miR-1, sja-miR-7-5p, sja-miR-36-3p) or host–parasite interaction (sja-bantam) [29]. Subsequent research has revealed that 24 miRNAs are also differentially expressed between the ten-day-old schistosomula from the non-permissive host *Microtus fortis* (*M. fortis*) and the susceptible host BALB/c mice [28]. Notably, some miRNAs show a similar expression trend in non-susceptible hosts and susceptible hosts. sja-miR-124-3p in worms recovered from non-susceptible hosts (Wistar rats, *M. fortis*, water buffalo) have a much higher expression level than that in susceptible hosts (BALB/c mice, yellow cattle), whereas sja-let-7, sja-miR-2b-5p, sja-miR-3493, sja-miR-125b and sja-miR-2c-5p are downregulated in worms from non-susceptible rodent hosts (Wistar rats, *M. fortis*) compared with those from susceptible rodent hosts (BALB/c mice) [28, 29]. Except for sja-miR-124-3p, the other four differentially expressed miRNAs in worms derived from water buffalo and yellow cattle were not well correlated with the result of previous study in different rodent hosts [28, 29]. These results might have arisen because the worms were from different definitive hosts, or the worms were at different developmental stages. Water buffalo, yellow cattle and rodent hosts provide a distinctive survival environment for *S. japonicum*, which might affect the miRNA expression of the worms. Schistosomes at different developmental stages usually possessed diverse miRNA expression profiles. These differentially expressed miRNAs and different expression trends in worms derived from different susceptible hosts may have important effects in regulating the growth, development and survival of schistosomes.

Like coding genes, miRNAs among different species have many orthologs, and some conserved miRNAs have been reported to have similar physiological functions [47, 48]. miR-124 is highly conserved and enriched in the central nervous system in many organisms, and is likely to play a significant role in central nervous system development and function [49–55]. Further research has found that miR-124 even plays an important role in the control of male sexual differentiation and behavior in *Drosophila* [56]. The sja-miR-124 in *S. japonicum* is highly expressed at the egg and cercariae stages of this parasite [19, 46], and is enriched in male worms [19]. Schistosomes have a complex nervous system and a well-developed sensory system that supports their response to a myriad of environmental stimuli at different life stages [31]. Free-living cercariae are equipped with abundant ciliated sensory papillae to sense changes in their environment (such as light, mechanical stimuli and temperature) [15, 31]. Thus, the complex nervous system and sensory system is critical to the survival and development of schistosomes. sja-miR-124-3p in schistosomes may play an important role in the regulation of the development of the sensory nervous system or male sexual differentiation, similarly to its roles in the model organisms (*Drosophila* and *Caenorhabditis elegans*). Male and female schistosomes are paired while they live in their definitive hosts under normal conditions. Males provide physical protection and release chemicals such as hormones, cholesterol, glucose and messengers that stimulate female growth, reproductive development, egg production and the maintenance of mature state [57–59]. Thus, the regulation of the development caused by sja-miR-124-3p in males may also affect the development of females indirectly and further lead to changes in their morphology and egg production. In addition, the expression trends of sja-miR-124-3p in worms from the less susceptible host water buffalo compared with the susceptible host yellow cattle are consistent with the results of our previous study in different susceptible rodent hosts. sja-miR-124-3p was the most significantly differentially expressed miRNA in ten-day-old schistosomula derived from different suitable rodent hosts (Wistar rats and BALB/c mice) [29]. Target gene prediction highlighted the potential regulatory function of sja-miR-124-3p in schistosomula development or growth. In the present study, a total of 70 mRNA transcripts were predicted to be potentially regulated by sja-miR-124-3p, such as mitochondrial import receptor subunit TOM40 homolog (Sjp_0016380), eukaryotic initiation factor 4A (Sjp_0042060), nuclear receptor subfamily 5, group A, member 2 (Sjp_0022650) and septin-7.1 (Sjp_0000290). GO analysis of these target genes revealed that they are mainly involved in protein transport, pharyngeal muscle development, apoptotic process, reproduction, regulation of dendrite morphogenesis, rRNA processing and vesicle-mediated transport. These results suggest that the differential expression of sja-miR-124-3p in *S. japonicum* derived from the two natural final hosts might be an important factor affecting the development and survival of schistosomes in their hosts.

miR-219 plays an important role in embryo development and has been shown to promote the differentiation of neural precursor cells. Overexpression of miR-219 induces obvious apoptosis in the head and tail of zebrafish [60–62]. Apoptosis is an important biological process in the schistosomulum and adult worm stages of *S. japonicum*. Several genes involved in apoptosis have been identified with a relationship to growth and development retardation in less susceptible hosts of *S. japonicum* [63, 64]. In the present study, the expression of sja-miR-219-5p in water buffalo-derived adults was higher than that in yellow cattle-derived worms. This may be one reason inducing the differential development of *S. japonicum* by affecting the apoptosis process. In addition, 28 potential target mRNAs of sja-miR-219-5p were preliminarily predicted through bioinformatic analyses. Among them, Hsc20 (Sjp_0008430) was validated by dual-luciferase reporter assay. Hsc20 is a J-type co-chaperone protein and regulates the ATPase and peptide-binding activity of Hsc66 [65, 66]. The exact function of Hsc20 in schistosomes has not been reported, but previous reports have indicated that Hsc20 and Hsc66 are important Hsp70 class molecular chaperones that are involved in the biogenesis of iron-sulfur proteins, ATP binding and hydrolysis [67, 68]. Hsp70 proteins play important roles in stress responses, protein processing and protein folding [68]. In the transformation process of cercariae to schistosomula and the subsequent growth in the final host, schistosomes are continuously stimulated by physiological factors, such as changes in temperature, salinity and the pH of the surrounding environment. Molecular chaperone systems, such as Hsc66 (Hsp70), J-proteins and other co-chaperone proteins, may play an important role in a variety of cellular processes. Consequently, sja-miR-219-5p may participate in apoptosis, ATP metabolism and stress responses by regulating the expression of Hsc20 and other molecules. It may indirectly affect stress responses, metabolism and other events of schistosomes and finally lead to differential development and survival of the worms from the two natural hosts.

Target prediction is an important pipeline used to learn about the possible functions of differentially expressed miRNAs. A previous study has reported that miRNAs typically bind to the 3′-UTRs of their mRNA targets, thus leading to transcript degradation [69]. Many miRNA target verification experiments have used 3′-UTR interaction sites, because many studies have shown miRNA effects with portions of the 3′-UTR [70]. However, in recent years, some studies have shown that the target sites are not limited to the 3′-UTR. There is increasing evidence that miRNAs bind to the 5′-UTR [71, 72] and the CDS [73–75] of target genes. For example, Forman et al. [75] have experimentally demonstrated that the let-7 miRNA directly targets the miRNA-processing enzyme Dicer within its coding sequence. Schnall-Levin et al. [76] have reported that conserved miRNA targeting in *Drosophila* is as widespread in coding regions as in the 3′-UTRs. mRNAs with several miRNA binding sites in the CDS regions can also effectively be degraded. In the present study, putative miRNA target sites of differentially expressed miRNAs were predicted against the full length of *S. japonicum* mRNA transcripts (5′-UTR, CDS or 3′-UTR), as reported in a previous *S. japonicum* miRNA study [17]. After repetitive refinement and verification of putative targets with their corresponding miRNAs by dual-luciferase reporter assays, sja-miR-219-5p was found to regulate the expression of Sjp_0008430 with the ‘seed region’ located in its CDS region.

After comprehensive analysis of the 268 predicted target genes potentially regulated by the five differentially expressed miRNAs obtained in this study, with the differentially expressed genes identified through a *S. japonicum*-specific microarray analysis in our previous study [15], 11 differentially expressed genes were found. These genes showed common expression trends in both detection techniques, thus implying that they might be key targets affecting the survival and development of *S. japonicum* in the two natural hosts. Further analysis revealed that these 11 targets have important biological functions; for example, septin-7.1, a member of the septin family, which is involved in vesicle trafficking and vesicle fusion, was found to be probably regulated by sja-miR-124-3p. Cytohesin-2, a guanine nucleotide-exchange factor for ARF, which activates ARF6, thereby regulating actin reorganization and membrane ruffling at the plasma membrane, is potentially targeted by sja-miR-3490. Eukaryotic initiation factor 4A is a highly conserved RNA-stimulated ATPase and helicase involved in the initiation of mRNA translation, which has been found to play an important role in ovule development and cell size homeostasis in *Arabidopsis* [77]. However, the available information was limited to make further correlation analysis between differentially expressed miRNAs and the differentially expressed proteins of worms derived from yellow cattle and water buffalo [16]. A total of 131 differentially expressed proteins were identified between worms from yellow cattle and water buffalo in our previous study, but only 49 differentially expressed proteins were annotated in the known database [16]. When the genome and protein function annotation of *S. japonicum* has been further refined and improved in the future, more studies need to be carried out to explain the correlation between the differentially expressed miRNAs and those of differentially expressed genes and proteins of *S. japonicum* isolated from water buffalo and yellow cattle.

## Conclusions

This study presents the first report on different miRNA expression profiles in adult *S. japonicum* isolated from the susceptible host yellow cattle and the less susceptible host water buffalo. Identifying differentially expressed miRNAs in *S. japonicum* from the two natural hosts is an important step toward understanding their potential roles in the survival and development of the parasite. These results may lay a foundation for further functional research on these miRNAs and provide a new basis for understanding the developmental mechanism of schistosomes and the interplay between schistosomes and their natural hosts.

## Additional files

**Additional file 1: Figure S1.** The work-flow of sequencing data analysis.

**Additional file 2: Table S1.** The information related to the precursor miRNAs of predicted miRNAs.

**Additional file 3: Table S2.** Sequences of the primers used for qRT-PCR and sja-miR-219-5p mimics or inhibitors used for dual-luciferase reporter assays. Sequences of the primers used for qRT-PCR of the five differentially expressed miRNAs.

**Additional file 4: Table S3.** Sequences of the primers used for qRT-PCR of the 11 differentially expressed targets.

**Additional file 5: Table S4.** Sequences of sja-miR-219-5p mimics or inhibitors and their corresponding controls used for dual-luciferase reporter assays.

**Additional file 6: Table S5.** Information on the 79 known miRNAs of *S. japonicum*, determined by sequencing and analysis.

**Additional file 7: Table S6.** Profiles of five differentially expressed known miRNAs of schistosomes derived from yellow cattle and water buffalo.

**Additional file 8: Table S7.** Summary of total miRNA expression profiles in adult *S. japonicum* isolated from yellow cattle and water buffalo.

**Additional file 9: Table S8.** Profiles of annotated novel miRNAs of schistosomes derived from yellow cattle and water buffalo.

**Additional file 10: Table S9.** Profiles of ten differentially expressed novel miRNAs of schistosomes derived from yellow cattle and water buffalo.

**Additional file 11: Table S10.** Primary analysis of the identified differentially expressed novel miRNAs (water buffalo/yellow cattle).

**Additional file 12: Figure S2.** Predicted secondary structures of novel miRNAs in *S. japonicum.* Dominant forms of the mature miRNAs are indicated in red. **a** SJC_S000996_20012_star@@sma-miR-8440-3p. **b** SJC_S027751_46535_star@@sma-miR-8468-5p. **c** SJC_S002031_35912_star@@sma-miR-8459-3p. **d** SJC_S000428_27179_mature@@rno-miR-489-3p. **e** SJC_S016027_44348_mature@@sma-miR-8480-5p.

**Additional file 13: Table S11.** Profiles of five known differentially expressed miRNAs and their targets.

**Additional file 14: Table S12.** Functional enrichment analysis of the predicted targeted genes. Biological process of targets in schistosomes of five known differentially expressed miRNAs.

**Additional file 15: Table S13.** Cellular components of targets in schistosomes of five known differentially expressed miRNAs.

**Additional file 16: Table S14.** Molecular function of targets in schistosomes of five known differentially expressed miRNAs.

**Additional file 17: Table S15.** KEGG pathway of targets in schistosomes of five known differentially expressed miRNAs.

## Abbreviations

PCR: polymerase chain reaction
RIN: RNA integrity number
GEO: Gene Expression Omnibus database
BWA: Burrows–Wheeler alignment
TMM: trimmed mean of M-values
UTR: untranslational regions
CDS: coding sequence
qRT-PCR: quantitative reverse transcription PCR
ECM: enriched in extracellular matrix
HEK 293T cell: human embryonic kidney 293T cell
GO: gene ontology
KEGG: Kyoto Encyclopedia of Genes and Genomes
