# Supplementary figures and images for: Comparative analysis of microRNA expression profiles of adult Schistosoma japonicum isolated from water buffalo and yellow cattle
Source: Parasit Vectors. 2019 May 2;12:196. doi: 10.1186/s13071-019-3450-7 (PMC6498558; doi:10.1186/s13071-019-3450-7)

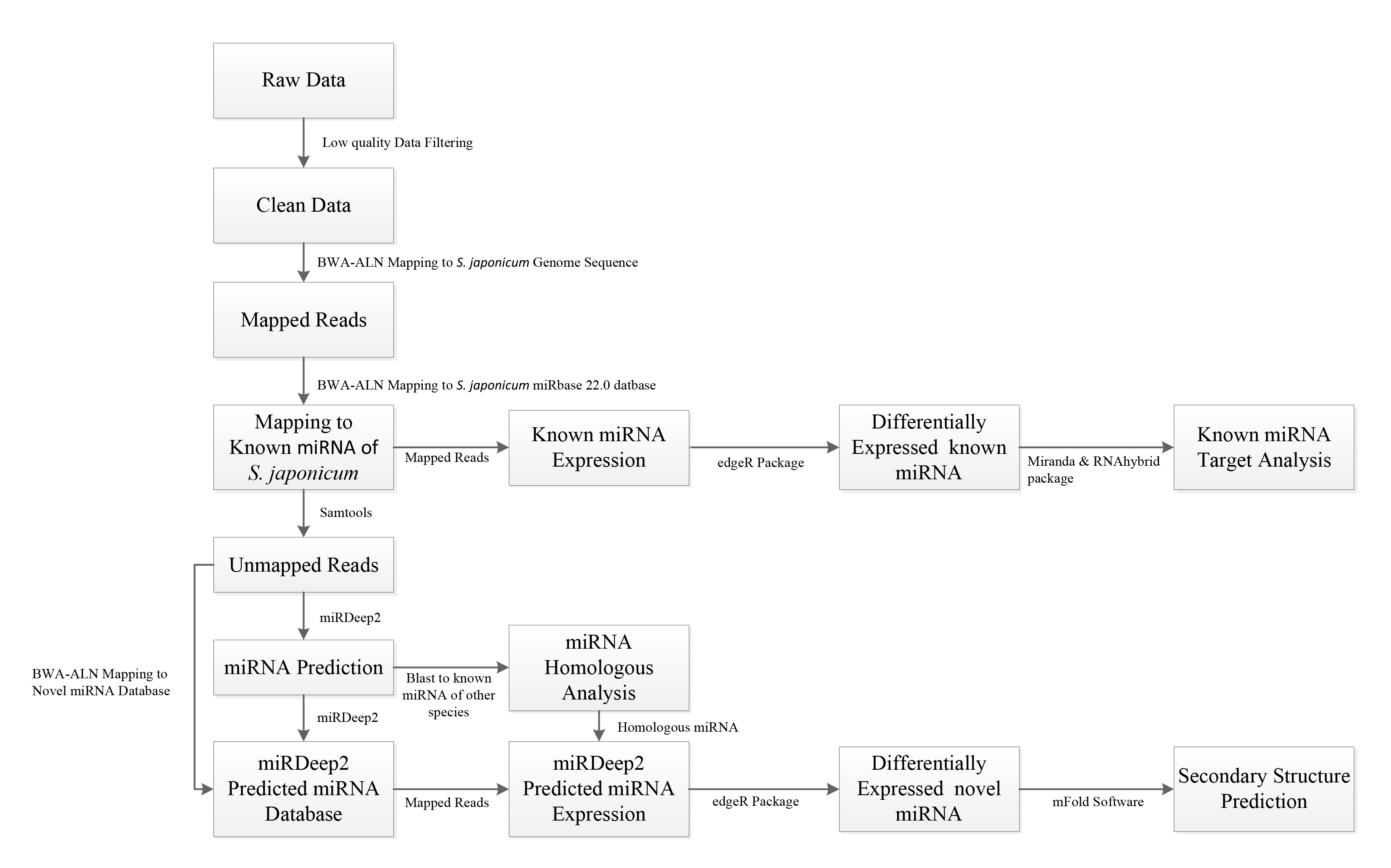

Supplement: Supplementary file 1 — Additional file 1: Figure S1. The work-flow of sequencing data analysis. [file 13071_2019_3450_MOESM1_ESM.tif]

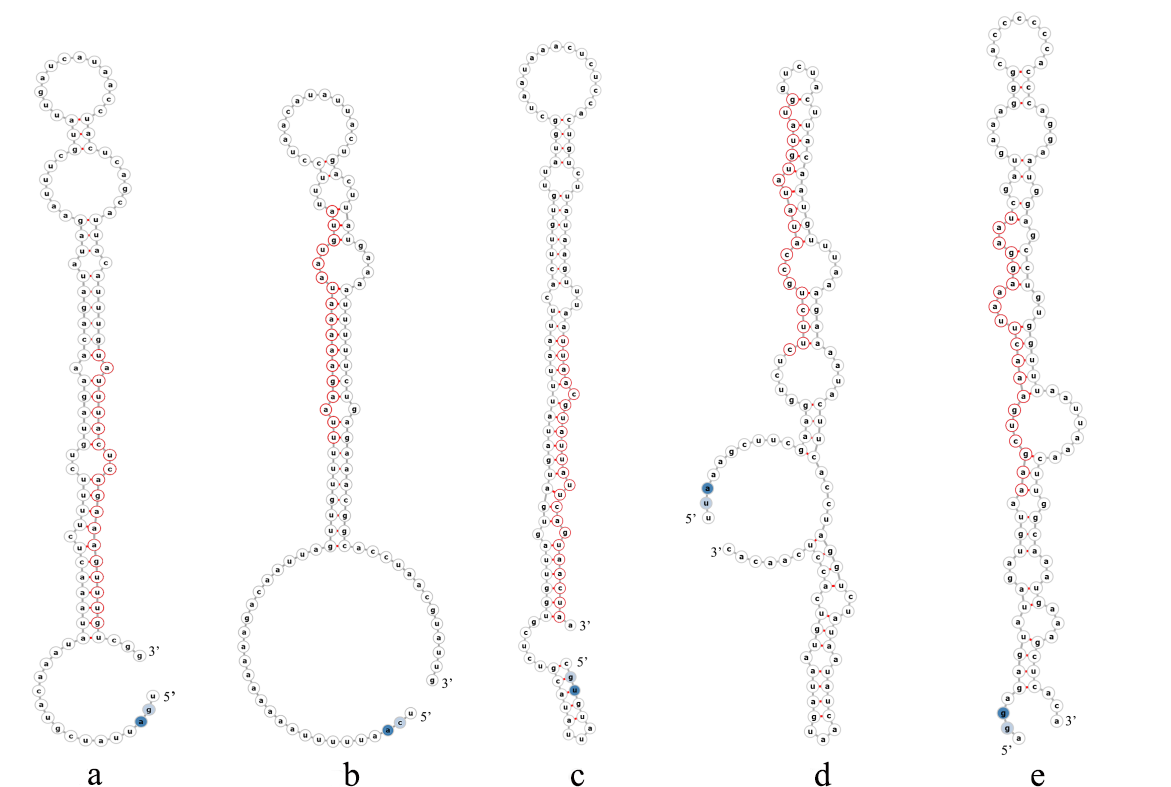

Supplement: Supplementary file 12 — Additional file 12: Figure S2. Predicted secondary structures of novel miRNAs in S. japonicum. Dominant forms of the mature miRNAs are indicated in red. a SJC_S000996_20012_star@@sma-miR-8440-3p. b SJC_S027751_46535_star@@sma-miR-8468-5p. c SJC_S002031_35912_star@@sma-miR-8459-3p. d SJC_S000428_27179_mature@@rno-miR-489-3p. e SJC_S016027_44348_mature@@sma-miR-8480-5p. [file 13071_2019_3450_MOESM12_ESM.tif]
